# A comparative analysis of early postoperative outcomes of autologous pericranium versus high-density polypropylene in cranial duraplasty in a tertiary care center in Central India

**DOI:** 10.1186/s41016-026-00440-y

**Published:** 2026-07-07

**Authors:** Shubham Gupta, Rakesh Gupta, Zafar Sheikh, Kapil Jain, Saksham Kumar

**Affiliations:** 1 Department of Neurosurgery, Sawai Man Singh Medical College and Hospital, Jaipur, Rajasthan India; 2 Department of Neurosurgery, M.G.M. Medical College and MYH Hospital, Indore, Madhya Pradesh India; 3 Department of Neurosurgery, Max Smart Super Speciality Hospital, Saket, New Delhi India

**Keywords:** Dural substitute, Pericranium, G-Patch, Cranial surgery, Duraplasty, Postoperative complications

## Abstract

**Background and objectives:**

Duraplasty is commonly required when primary dural closure is not feasible during cranial surgery. Although both autologous and synthetic dural substitutes are widely used, high-quality prospective data comparing their early postoperative safety remain limited. This study aimed to compare early postoperative complication rates following cranial duraplasty using autologous pericranium versus high-density polypropylene (G-Patch).

**Methods:**

This prospective randomized comparative study enrolled 121 consecutive patients undergoing cranial duraplasty at a tertiary care center between January and December 2021. Following exclusion of patients who died within 30 days postoperatively, 100 patients (50 per group) were included in the final analysis. Patients were allocated to receive either autologous pericranium (*n* = 50) or high-density polypropylene (G-Patch) (*n* = 50) using a standardized surgical technique. The primary outcome was the occurrence of any postoperative complication within 30 days, including cerebrospinal fluid leak, wound infection, and subdural empyema. Effect estimates were expressed as odds ratios (OR) with 95% confidence intervals (CI), and a limited adjusted analysis was performed as a sensitivity assessment.

**Results:**

Traumatic brain injury and intracranial space-occupying lesions were the most common indications for surgery. Most patients in both groups had an uneventful postoperative course. Early postoperative complications occurred in 26% of patients in the synthetic graft group and 12% in the autologous group. The unadjusted odds ratio for any complication with synthetic graft use was 2.58 (95% CI: 0.88–7.54; *p* = 0.08). After adjustment for primary diagnosis, graft type remained a non-significant predictor of complications. No consistent associations were observed between postoperative complications and comorbidities or substance use in exploratory analyses.

**Conclusion:**

In this prospective randomized cohort, both autologous pericranium and high-density polypropylene demonstrated acceptable short-term safety profiles. However, given the limited sample size, imbalance in underlying diagnoses, and short follow-up, these findings should be interpreted with caution and should not be considered evidence of equivalence between graft materials. Further studies with larger sample sizes, stratified analyses, and long-term outcomes are required.

**Trial registration:**

This investigator-initiated pragmatic randomized study was not prospectively registered in a clinical trial registry.

**Supplementary Information:**

The online version contains supplementary material available at 10.1186/s41016-026-00440-y.

## Background

Achieving satisfactory dural closure after cranial surgery remains a key technical consideration, particularly in the presence of large dural defects, trauma, or dural resection. Inadequate dural repair may be associated with cerebrospinal fluid (CSF) leakage, infection, pseudomeningocele, and prolonged hospitalization [1, 2]. When primary dural closure is not feasible, duraplasty is performed to restore dural integrity and reduce postoperative morbidity [3].

A wide range of dural substitutes is currently available, broadly categorized as autologous, xenogeneic/allogeneic, and synthetic materials. Autologous grafts, such as pericranium, are widely regarded as the reference standard because of their excellent biocompatibility, low risk of immunogenic reaction, and favorable integration with the native dura [3, 4]. However, their availability may be limited in patients with prior surgery, scalp trauma, or compromised tissue quality. Synthetic substitutes, including high-density polypropylene (G-Patch), offer immediate availability and consistent material properties, but concerns persist regarding foreign-body reaction, infection risk, and long-term biological integration [5].

Traditionally, the ability to achieve a watertight dural closure has been considered an important attribute of an ideal dural substitute, particularly in infratentorial procedures and in cases with elevated CSF pressure [6]. However, emerging evidence suggests that strict watertight closure may not be mandatory in selected supratentorial surgeries, where non-watertight closure has been shown to result in acceptable outcomes without increased complication rates [7]. This has led to ongoing debate regarding the relative importance of watertight closure versus other graft characteristics, such as biocompatibility and tissue integration, depending on surgical location and context.

Despite widespread clinical use of both autologous and synthetic dural substitutes, high-quality prospective comparative data remain limited. Most available studies are retrospective, focus on specific pathologies, or evaluate heterogeneous graft materials, making it difficult to draw definitive conclusions regarding comparative safety and complication profiles. Furthermore, the influence of patient-related factors and surgical variables on postoperative outcomes is inconsistently addressed in the literature.

In this context, we conducted a prospective randomized comparative study comparing autologous pericranium and high-density polypropylene (G-Patch) for cranial duraplasty. The primary objective was to compare early postoperative complication rates between the two graft types. Secondary exploratory analyses examined the association between selected patient-related factors and postoperative outcomes.

## Methods

This study was designed as a prospective randomized comparative study. All consecutive eligible patients undergoing cranial duraplasty at our center during the study period (January–December 2021) were included in the study. This study aimed to evaluate the outcomes of two different dural substitutes: autologous pericranium and non-autologous high-density polypropylene.

**Fig. 1:**
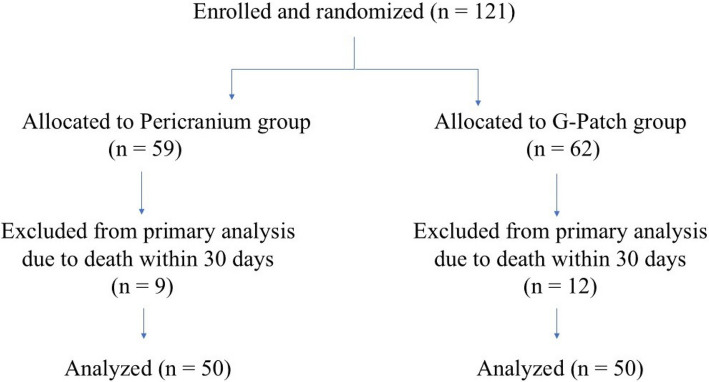
CONSORT-style flow diagram showing patient enrollment, randomization, exclusions due to early postoperative mortality, and final analysis

The study is reported in accordance with CONSORT guidelines for randomized studies. A patient flow diagram illustrating enrollment, allocation, and analysis is provided (Fig. 1).

For the inclusion criteria, patients were eligible for the study if they were undergoing cranial neurosurgical procedures requiring dural repair or substitution. Additionally, individuals were included if they presented with dural defects unsuitable for primary closure, thereby necessitating dural substitution. Crucially, all patients were required to provide informed consent for participation in the study before enrollment.

Regarding the exclusion criteria, several factors rendered patients ineligible to participate in the research. These included trauma patients presenting with open, potentially infected wounds, as well as those who were immunocompromised. Patients undergoing a re-exploration procedure were also excluded. Furthermore, individuals with an active systemic infection or a local scalp infection at the surgical site could not be enrolled. Finally, participants with a known allergy or hypersensitivity to synthetic dural substitute materials were excluded from the study.

The study was conceived as an exploratory comparison; no a priori sample size or power calculation was performed. The final sample size reflects the number of eligible cases treated during the study period. A total of 121 eligible patients were randomized using a computer-generated allocation sequence. Group A received autologous pericranium, while Group B received G-Patch: high-density polypropylene (Manufacturer: Surgiwear Ltd., India). Following exclusion of patients who died within 30 days postoperatively, 100 patients (50 in each group) were included in the final analysis. The synthetic grafts were procured through routine institutional supply, and no external funding or industry support was involved. Allocation concealment was ensured using sealed opaque envelopes, opened only at the time of surgery. Randomization was intended to reduce selection bias; however, residual imbalance and confounding remain possible given the modest sample size and heterogeneous cohort. Blinding was not feasible due to the nature of the surgical intervention. Surgical cases were categorized as supratentorial or infratentorial based on lesion location; however, the study was not powered to perform stratified comparative analyses between these subgroups.

All procedures were performed under general anesthesia by a single experienced neurosurgeon using a standardized operative protocol to minimize technical variability.

A scalp incision appropriate to the underlying pathology was made, followed by reflection of the scalp flap with preservation of the pericranium whenever feasible. Hemostasis was achieved using bipolar cautery and bone wax as required. After craniotomy and completion of the primary intracranial procedure, the dura was assessed for the suitability of primary closure.

**Fig. 2:**
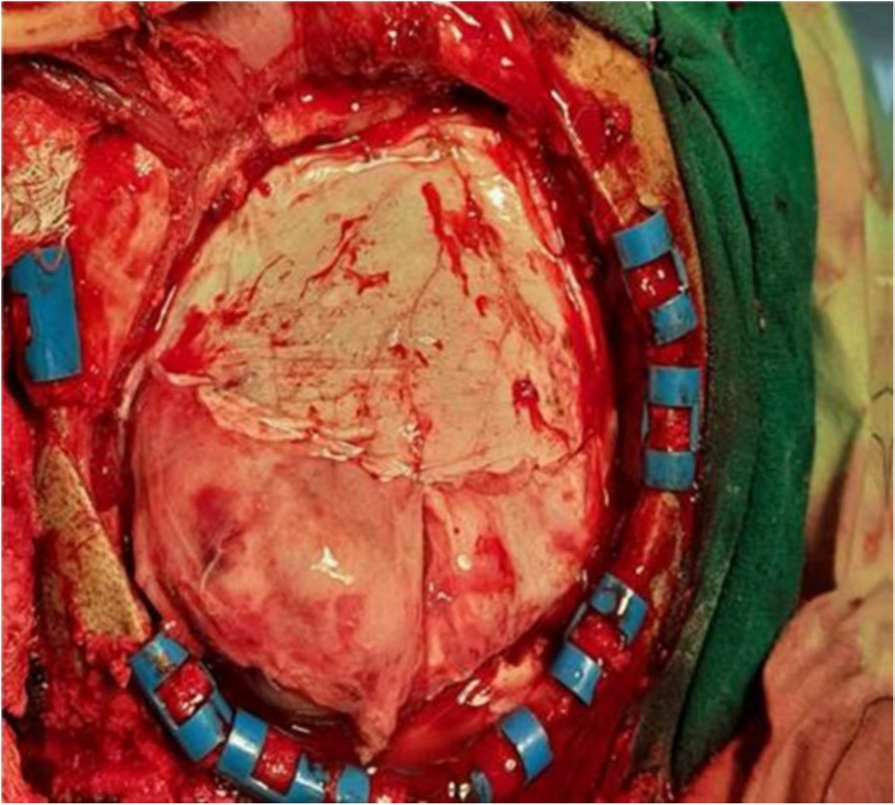
Augmentation duraplasty with pericranium in space-occupying lesion excision

**Fig. 3:**
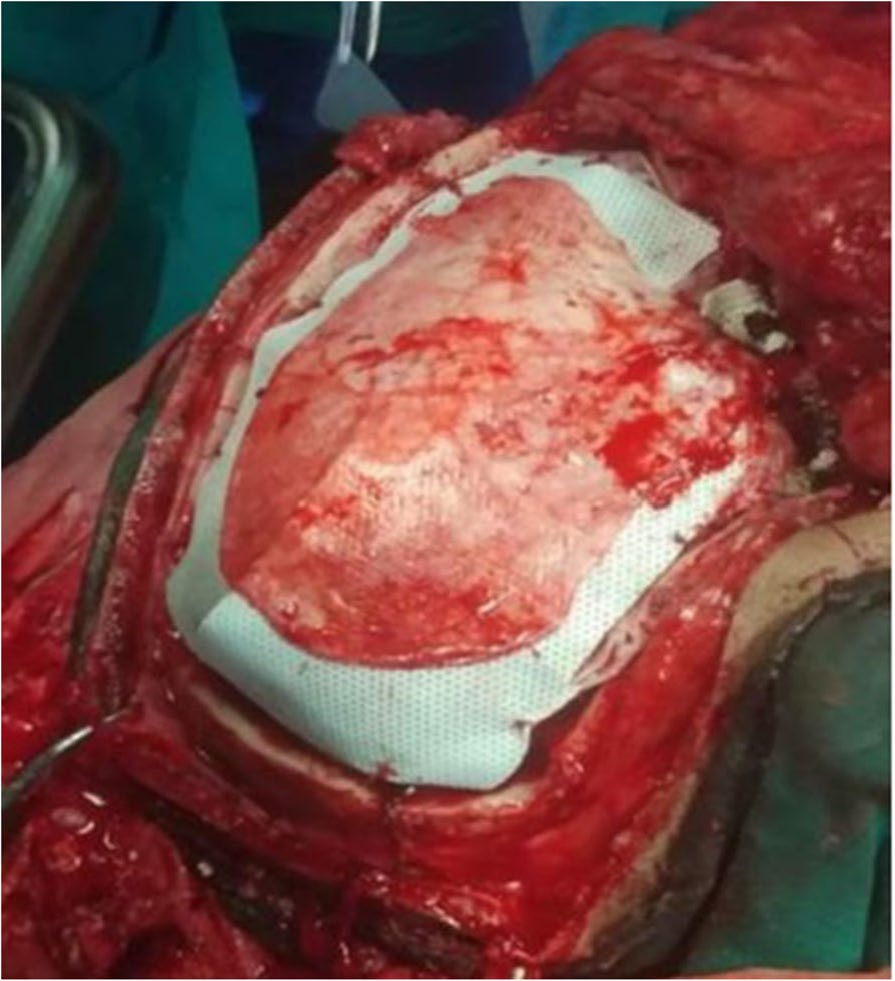
Augmented duraplasty using high-density polypropylene dural patch (G-Patch) in decompressive craniectomy

In patients randomized to autologous grafting, a galea-pericranial graft was harvested from the reflected scalp flap, tailored to the size of the dural defect, and kept moist in normal saline until implantation. In patients allocated to synthetic grafting, the high-density polypropylene (G-Patch) was trimmed to the required size and prepared according to the manufacturer’s recommendations. The graft was sutured to the native dura using either interrupted or continuous non-absorbable sutures to achieve a watertight closure (Figs. 2 and 3). Additional sutures were placed as necessary to eliminate gaps at the graft–dura interface. The wound was closed in layers, and a sterile dressing was applied.

Postoperative daily wound examinations were performed. Patients were monitored for complications, including wound infection, CSF leak, subcutaneous CSF collection, meningitis, subdural empyema, wound dehiscence, and bone flap osteitis. After neurological stabilization, patients were discharged and followed up for a minimum period of 30 days. No patients were lost to follow-up during this period. Patients who did not survive the immediate postoperative period (< 30 days) were excluded from outcome analysis, as postoperative complications could not be reliably assessed.

Postoperative complications were assessed daily during hospitalization and at the 30-day follow-up. Each complication was explicitly defined as follows:

- Wound infection was defined as the presence of local signs of infection at the surgical site (erythema, discharge, or wound dehiscence) with or without microbiological confirmation requiring antibiotic therapy or surgical intervention [8].
- CSF leak was defined as clinically evident leakage of CSF through the surgical wound or incision site [9].
- Subcutaneous CSF collection (pseudomeningocele) was defined as a localized fluctuant swelling at the surgical site consistent with CSF accumulation without external leakage [10].
- Meningitis was defined based on clinical features (fever, neck stiffness, altered sensorium) with supportive cerebrospinal fluid findings suggestive of infection [11].
- Subdural empyema was defined as a collection of purulent material in the subdural space confirmed radiologically or intraoperatively [12].
- Bone flap osteitis was defined as infection involving the bone flap or surrounding cranial bone, confirmed clinically and/or radiologically, with or without the need for surgical debridement [13].
- Wound dehiscence was defined as partial or complete separation of the surgical wound edges requiring medical or surgical management [14].

The study was conducted in accordance with the principles outlined in the Declaration of Helsinki. The patients gave informed consent. Ethical clearance for this study was obtained from the Institutional Ethics Committee, vide letter number EC/MGM/NOV-20/129.

Statistical analysis: Categorical variables were presented in numbers and percentages (%), and continuous variables were presented as mean ± SD. Quantitative variables were compared using an unpaired *t*-test between the two groups. Qualitative variables were compared using the chi-square test/Fisher’s exact test. A *p*-value of < 0.05 was considered statistically significant. The data were entered in an MS Excel spreadsheet, and analysis was done using Statistical Package for Social Sciences (SPSS) version 21.0. To compare the primary endpoint of “any postoperative complication,” we calculated the odds ratio (OR) with a 95% confidence interval (CI). A limited multivariable logistic regression analysis was performed as a sensitivity assessment; however, given the small number of events, results should be interpreted with caution.

Due to low event counts in several complication subcategories and subgroup variables (comorbidities and substance use), these analyses were considered exploratory. Inferential statistical testing was avoided where assumptions for validity were not met, and such data are presented descriptively. Detailed tables are provided in the supplementary material.

## Results

A total of 121 patients were enrolled and randomized during the study period, with 59 patients allocated to the pericranium group and 62 patients allocated to the G-Patch group. Following randomization, 21 patients died within 30 days postoperatively (pericranium group: 9; G-Patch group: 12) and were excluded from the primary complication analysis. The final analyzed cohort, therefore, consisted of 100 patients, with 50 patients in each treatment group (Table 1). All patients included in the final analysis completed the 30-day follow-up, with no losses to follow-up. Early postoperative deaths were predominantly observed in patients with severe traumatic brain injury and malignant intracranial lesions and were attributed to progression of the primary neurological disease rather than graft-related complications. A sensitivity analysis was performed by including early postoperative deaths as part of a composite adverse outcome (any postoperative complication or death). Using this composite endpoint, adverse outcomes occurred in 25 of 62 patients in the G-Patch group (12 early deaths and 13 postoperative complications) and 15 of 59 patients in the pericranium group (9 early deaths and 6 postoperative complications). The odds ratio was 1.98 (95% CI: 0.91–4.30; *p* = 0.08). This analysis did not materially alter the overall interpretation of the study findings.

**Table 1:** Demographic and clinical profile of patients (*N* = 100)

Sociodemographic and clinical parameters	G-patch	Pericranium	Total
Age group (years)			
0–14	1	9	10
15–29	14	10	24
30–44	17	10	27
45–59	11	14	25
60–74	7	7	14
Gender			
Male	30	31	61
Female	20	19	39
Diagnosis			
Head injury	40	18	58
Tumour	8	25	33
Miscellaneous	0	5	5
Non-traumatic bleed	2	2	4
Distribution by surgical location			
Supratentorial	46	47	93
Infratentorial	4	3	7
Substance abuse			
Nil	28	39	67
Alcohol	12	6	18
Smoking	6	3	9
Tobacco	4	2	6
Comorbidities			
Nil	43	39	82
Hypertension	4	3	7
DM	1	5	6
Asthma	1	1	2
CAD	1	0	1
CKD	0	1	1
Hepatitis B	0	1	1

The most common age group was 30–44 years (27%), followed by 15–29 years (24%) and 45–59 years (25%). Among the 100 patients, 61% were male, and 39% were female. Traumatic brain injury was the most common diagnosis (58%), while brain tumors accounted for 33% of cases. Most of the patients (82%) had no comorbidities; hypertension (7%) and diabetes mellitus (6%) were the most frequent among those with comorbid conditions.

Regarding substance use, most patients had no history of substance abuse (67%). Among those who did, alcohol use was the most common (18%), followed by smoking (9%) and tobacco chewing (6%). Dural substitution was equally performed using G-Patch and autologous pericranium in 50% of patients each.

Table 2 shows the distribution of postoperative complications according to the type of dural substitute used in our study: high-density polypropylene versus pericranium. The majority of patients in both groups experienced no complications (74% in the polypropylene group and 88% in the pericranium group). Wound infection was the most common complication, occurring in 16% of patients with high-density polypropylene and 8% with pericranium. Other complications, such as subcutaneous CSF collection, CSF leak, subdural empyema, meningitis, bone flap osteitis, and wound dehiscence, were observed at low frequencies across both groups.

**Table 2:** Distribution of postoperative complications by type of dural substitute used

Complication	High-density polypropylene (*n* = 50)	Pericranium (*n* = 50)
Nil (no complications)	37	44
Wound infection	8	4
Subcutaneous CSF collection	4	0
CSF leak	2	1
Subdural empyema	1	2
Meningitis	1	0
Bone flap osteitis	1	0
Wound dehiscence	0	2

To address the baseline imbalance in surgical indications, a post-hoc exploratory analysis was conducted specifically within the traumatic brain injury (TBI) subgroup (*n* = 58). Within this high-risk cohort, the complication rate was 15% for the high-density polypropylene group compared to 14% for the autologous pericranium group; however, this exploratory analysis is limited by small sample size and should be interpreted with caution. The distribution of surgical location was comparable between the two groups. In the pericranium group, 47 patients underwent supratentorial procedures and 3 underwent infratentorial procedures. In the G-Patch group, 46 patients had supratentorial procedures, and 4 had infratentorial procedures. These data are presented descriptively, and no stratified analysis was performed due to sample size limitations.

**Fig. 4:**
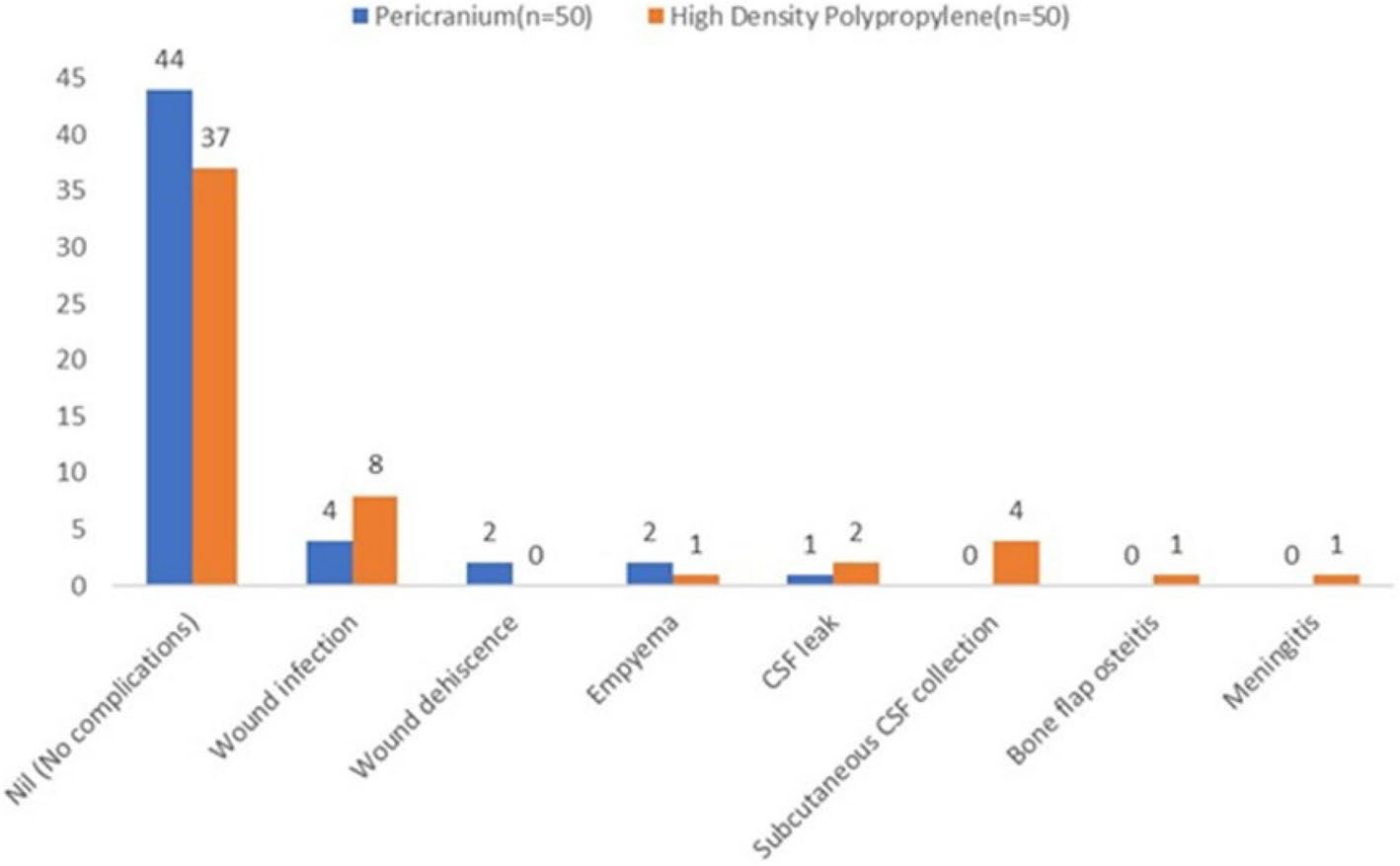
Post-op complication comparison between autologous and high-density polypropylene dural substitute

Figure 4 shows that the pericranium group had a higher rate of uneventful recovery (88%) compared to the high-density polypropylene group (74%). The overall complication rate was 26% (13/50) in the high-density polypropylene group and 12% (6/50) in the autologous pericranium group. The unadjusted odds ratio for any complication in the synthetic group was 2.58 (95% CI: 0.88–7.54, *p* = 0.08). When adjusting for primary diagnosis in a logistic regression model, the choice of material remained a non-significant predictor of complications (Adjusted OR: 1.84, 95% CI: 0.54–6.27, *p* = 0.33), whereas the diagnosis of TBI showed a stronger, though non-significant, association with complication risk.

Analyses examining the association between postoperative complications and patient-related factors, including comorbidities and substance use, were limited by low event counts. These findings are therefore presented descriptively and are provided in Supplementary Tables A and B. Descriptive analyses did not demonstrate any clear trends; however, interpretation is limited by sparse event counts and overlapping patient categories.

Supplementary Table A summarizes the distribution of postoperative complications according to various patient comorbidities, including asthma, coronary artery disease (CAD), chronic kidney disease (CKD), diabetes mellitus (DM), hepatitis B, and hypertension. The majority of patients without complications had no documented comorbidities (79.0%), while smaller proportions had individual comorbidities. Wound infection occurred predominantly in patients without comorbidities (91.7%), with only a single case observed in a hypertensive patient. Other complications, such as subcutaneous CSF collection, CSF leak, subdural empyema, wound dehiscence, meningitis, and bone flap osteitis, were exclusively observed in patients without any recorded comorbidities. The distribution of postoperative complications was also examined in relation to substance abuse, including alcohol use, smoking, and tobacco chewing, as detailed in supplementary Table B. Most patients without complications did not have a history of substance abuse (65.4%), while smaller proportions reported alcohol (18.5%), smoking (11.1%), or tobacco use (4.9%). Wound infections and other complications occurred primarily in patients without substance abuse, although isolated cases were observed among alcohol and tobacco users. Descriptive analyses of postoperative complications according to comorbidity and substance-use history are presented in the supplementary tables. Given the sparse event counts and overlapping patient categories, no inferential statistical conclusions were drawn from these analyses.

## Discussion

Duraplasty involves repairing dural defects by applying either autologous (natural) or synthetic (non-autologous) grafts to restore dural integrity. The first reported use of dural substitutes dates back to 1895, by Robert Abbe [3, 15], and since then, various materials have been utilized for dural reconstruction.

An ideal dural substitute should provide a watertight seal, closely mimic the natural dura mater’s mechanical properties, be cost-effective, easy to handle when wet, minimize inflammation and scarring, and promote neodura formation [3, 15, 16]. Over the past five decades, autologous grafts such as pericranium, fascia lata, and temporalis fascia have been widely used [15]. These grafts are inexpensive, non-immunogenic, and integrate well with native dura but may be limited by availability and require an additional surgical site for harvest.

Cadaveric dura, first described by Campbell et al. [17], offers abundant graft material but carries risks such as transmission of Creutzfeldt-Jakob disease or bovine spongiform encephalopathy, limiting its use. Synthetic dural substitutes, made from polymers like high-density polypropylene, provide an inert alternative with good strength, elasticity, and malleability. However, they may provoke foreign body reactions, inflammation, and lack of vascularization or epithelial fusion seen with autologous grafts [18, 19].

The choice of a dural substitute depends on factors such as defect size, surgical approach, availability, and cost. The use of different dural substitutes depends on the indications for duraplasty. In a recent article, it was determined that the most common indication for duraplasty was tumor resection (53% cases), which was covered using synthetic grafts [15]. In contrast, around 60% of duraplasty cases in our cohort were decompressive craniectomies for traumatic brain injury, with excision of space-occupying lesions being the second most common indication.

The duration to harvest pericranium was minimal and did not affect operative time compared to the immediately available synthetic graft. Both grafts provided sufficient material to close large dural defects effectively. Although watertight dural closure has traditionally been emphasized, especially in posterior fossa surgery, evidence suggests that in selected supratentorial procedures, a non-watertight closure may be acceptable [7, 20]. In our practice, we aimed for watertight closure in all cases to standardize technique and minimize confounding, particularly given the heterogeneous cohort. A study by Sabatino et al. [21] demonstrated that during supratentorial craniotomies, adequate autologous grafts could be easily harvested to cover any dural defect, regardless of size. Although G-Patch is typically more expensive than biological grafts, its availability through institutional support ensured that no additional financial burden was transferred to the patients, making it a more economical choice from the hospital’s perspective.

In the present study, complication rates were higher in the synthetic graft group, although the difference was not statistically significant. In a study by Malliti et al. [22], the use of neuropatch (synthetic substitute) was associated with increased deep wound infection (15% vs. 5%, *p* = 0.06) and CSF leaks (13% vs. 1.6%, *p* < 0.05). This was attributed to a foreign-body reaction to the implanted synthetic dural substitute. However, the difference in complication rate between group A and B was not statistically significant. Wound infection was the most frequent complication, followed by CSF leak and subcutaneous CSF collection. These results align with previous studies, which generally report no significant differences in complication rates between autologous and synthetic dural substitutes, though some have noted a trend favoring autologous grafts [4, 5, 15, 23]. Early postoperative deaths were predominantly observed in patients with severe traumatic brain injury and malignant intracranial lesions and were attributed to progression of the primary neurological disease rather than duraplasty-related complications; however, their exclusion may still introduce survivorship bias. Nevertheless, a sensitivity analysis incorporating early deaths into a composite adverse outcome did not materially alter the overall findings.

Beyond the choice of graft material, dural substitutes have been explored as adjuncts to reduce postoperative complications. While direct comparative trials are lacking, different studies highlight distinct complication profiles between synthetic and autologous dural substitutes. In a meta-analysis by Shah et al. [24], no study demonstrated significant inferiority of the synthetic dural sealants compared to autologous graft in terms of complications and infection rate. In a study by Graziano et al. [25], the fully autologous sealant Vivostat demonstrates exceptional biocompatibility with a 0% infection rate and a very low 1.5% CSF leak rate. While not directly comparable to dural substitutes, these findings suggest that biological adjuncts may complement dural repair strategies, particularly in reducing CSF-related complications.

Unlike prior studies [5] that focused on a single pathology, such as traumatic brain injury, the present study included patients undergoing cranial duraplasty for diverse indications. However, this heterogeneity was accompanied by an imbalance in underlying diagnoses between the two graft groups, with traumatic brain injury predominating in the G-Patch group and tumor-related procedures more frequent in the pericranium group. As etiology, defect characteristics, contamination risk, and adjuvant therapies are known to influence postoperative complication rates, this imbalance introduces potential confounding by indication. As postoperative risk profiles differ between conditions such as traumatic brain injury and tumor surgery, direct comparisons between groups should be interpreted with caution. The absence of a priori sample size calculation and the relatively small sample size limit the statistical power of the study to detect clinically meaningful differences between groups. Therefore, the lack of statistically significant differences should not be interpreted as evidence of equivalence. Similar to our study, Abla AA et al. [4], in their literature review, did not support the superiority of either autologous or non-autologous grafts when duraplasty is employed in Chiari decompression surgery.

Cost considerations are important when selecting dural substitutes, particularly in tertiary care and resource-constrained settings [21, 25]. Autologous pericranium avoids material costs but may be unavailable in some cases, whereas synthetic grafts, such as high-density polypropylene, offer immediate availability at a higher material cost. Although this study was not designed to evaluate cost-effectiveness, the comparable short-term safety profiles observed suggest that graft selection may also be influenced by institutional resources and cost considerations. While the duration of hospital stay may reflect duraplasty success, it is influenced by multiple factors, including the patient’s neurological status and systemic complications such as respiratory infections [26, 27]. Although supplementary descriptive analyses explored postoperative complications according to comorbidity and substance-use history, the limited number of events and overlapping patient categories preclude meaningful inferential interpretation.

### Limitations

This study has several important limitations. Although the prospective design strengthens data collection, the sample size was modest and not powered for stratified or multivariable analyses. There was an imbalance in underlying surgical indications between groups, with traumatic brain injury more common in the synthetic graft group and tumor-related procedures more frequent in the pericranium group, introducing potential confounding by indication and limiting direct comparability. Although supratentorial and infratentorial duraplasty differ in anatomical and surgical considerations, the present study was not powered to evaluate location-specific outcomes, which limits inference regarding graft performance across these compartments. However, due to the lack of a statistically significant difference in complications, definitive conclusions regarding comparative performance cannot be drawn.

The cohort predominantly included cranial procedures with a relatively low risk of high-flow CSF leakage and pseudomeningocele formation. Therefore, the findings may not be generalizable to higher-risk settings such as skull base surgery, transsphenoidal approaches [28], cranialization procedures [29], or spinal dural repair [30], where CSF dynamics and closure requirements differ significantly. The 30-day follow-up period captures early postoperative outcomes but does not reflect long-term graft integration or delayed complications. In this analysis, no intraoperative variables, neurological recovery, functional status, or patient-reported quality-of-life outcomes were assessed. As these outcomes are integral to the comprehensive evaluation of dural substitute performance, their absence restricts the conclusions to short-term safety only. Although early deaths were attributed to the underlying pathology, their exclusion may introduce survivorship bias and affect complication estimates. Additionally, the study was not prospectively registered in a clinical trial registry.

Larger studies with longer follow-up, indication-specific analyses, and functional outcome measures are required to define the comparative effectiveness of dural substitutes better.

## Conclusion

This prospective randomized study evaluated the short-term safety of autologous pericranium and high-density polypropylene (G-Patch) for cranial duraplasty in a heterogeneous cohort. No statistically significant difference in early postoperative complication rates was observed between the two groups, although a higher number of complications occurred in the synthetic graft group. However, given the limited sample size, short follow-up, imbalance in underlying diagnoses, and absence of stratified analyses, these findings should be interpreted with caution and should not be considered evidence of equivalence between graft materials across different clinical scenarios.

Both grafts demonstrated acceptable short-term safety within the studied cohort. Further studies with larger sample sizes, indication-specific analyses, and incorporation of functional outcomes are required to better define the comparative performance of dural substitutes.

## Supplementary Information

Supplementary Material 1.

Supplementary Material 2.

## Data Availability

No datasets were generated or analysed during the current study.

## Abbreviations

CSF: Cerebrospinal fluid
DM: Diabetes mellitus
CAD: Coronary artery disease
CKD: Chronic kidney disease
